# Characterization of Hymenopteran Parasitoids of *Aphis fabae* in An African Smallholder Bean Farming System Through Sequencing of COI ‘Mini-barcodes’

**DOI:** 10.3390/insects10100331

**Published:** 2019-10-02

**Authors:** Prisila A. Mkenda, Patrick A. Ndakidemi, Philip C. Stevenson, Sarah E. J. Arnold, Steven R. Belmain, Maneno Chidege, Geoff M. Gurr, Victoria C. Woolley

**Affiliations:** 1 Department of Sustainable Agriculture, Biodiversity and Ecosystems Management, The Nelson Mandela African Institution of Science and Technology, Arusha PO Box 447, Tanzania; mayoprisca@gmail.com (P.A.M.); patrick.ndakidemi@nm-aist.ac.tz (P.A.N.); 2 Graham Centre for Agricultural Innovation, Charles Sturt University, PO Box 883, Orange, NSW 2800, Australia; GGurr@csu.edu.au; 3 Natural Resources Institute, University of Greenwich, Chatham Maritime, Kent ME4 4TB, UK; P.C.Stevenson@greenwich.ac.uk (P.C.S.); S.E.J.Arnold@greenwich.ac.uk (S.E.J.A.); S.R.Belmain@greenwich.ac.uk (S.R.B.); 4 Royal Botanic Gardens, Kew, Richmond Surrey, TW9 3AB, UK; 5 Department of Research, Plant Protection Division, Tropical Pesticide Research Institute (TPRI), Arusha PO Box 3024, Tanzania; maneno.chidege@tpri.go.tz; 6 Institute of Applied Ecology, Fujian Agriculture and Forestry University, Fuzhou 35002, China

**Keywords:** primary parasitoids, hyperparasitoids, *Aphidius colemani*, *Phaseolus vulgaris*, natural pest regulation, DNA barcoding

## Abstract

Parasitoids are among the most frequently reported natural enemies of insect pests, particularly aphids. The efficacy of parasitoids as biocontrol agents is influenced by biotic and abiotic factors. For example, hyperparasitoids can reduce the abundance of the primary parasitoids as well as modify their behavior. A field study was conducted at three contrasting elevations on Mount Kilimanjaro, Tanzania, to identify the parasitoids of aphids in smallholder bean farming agroecosystems. Sentinel aphids (*Aphis fabae*) on potted bean plants (*Phaseolus vulgaris*) were exposed in 15 bean fields at three elevations for 2 days. The sentinel aphids were then kept in cages in a greenhouse until emergence of the parasitoids, which were collected and preserved in 98% ethanol for identification. Of the 214 parasitoids that emerged from sentinel aphids, the greatest abundance (44.86%) were from those placed at intermediate elevations (1000–1500 m a.s.l), compared to 42.52% from the lowest elevations and only 12.62% from the highest elevation farms. Morphological identification of the parasitoids that emerged from parasitized aphids showed that 90% were *Aphidius* species (Hymenoptera: Braconidae: Aphidiinae). Further characterization by sequencing DNA ‘mini-barcodes’ identified parasitoids with ≥99% sequence similarity to *Aphidius colemani*, 94–95% sequence similarity to *Pachyneuron aphidis* and 90% similarity to a *Charipinae* sp. in the National Center for Biotechnology Information (NCBI) database. These results confidently identified *A. colemani* as the dominant primary aphid parasitoid of *A. fabae* in the study area. A *Pachyneuron* sp., which was most closely related to *P. aphidis*, and a *Charipinae* sp. occurred as hyperparasitoids. Thus, interventions to improve landscapes and farming practice should monitor specifically how to augment populations of *A. colemani*, to ensure any changes enhance the delivery of natural pest regulation. Further studies are needed for continuous monitoring of the hyperparasitism levels and the dynamics of aphids, primary parasitoids, and secondary parasitoids in different cropping seasons and their implications in aphid control.

## 1. Introduction

Common bean (*Phaseolus vulgaris* L.) is a popular food legume in Africa and Latin America [1]. It is the major source of dietary proteins among low-income households in these areas due to the expense of animal protein sources like fish, milk, eggs and meat. In Africa, and particularly Tanzania, beans are produced by resource-poor farmers with very limited inputs mainly on small scale and marginal farms. Common bean production is constrained by several environmental, climatic and agronomic factors, with insect pests being the most profound [2,3]. Major field insect pests of common beans in Northern Tanzania include aphids (Hemiptera: Aphididae), bean foliage beetle (Coleoptera: Chrysomelidae: *Ootheca* sp.)*,* blister beetles (Coleoptera: Meloidae), pod borers (Lepidoptera: Crambidae), whiteflies (Hemiptera: Aleyrodidae), and thrips (Thysanoptera). Aphids, *Aphis fabae* Scopoli (Hemiptera: Aphididae), are an abundant and destructive pest of common beans and can cause damage by direct feeding, honey dew production, and transmission of diseases [4]. Historically, *A. fabae* has been controlled using synthetic pesticides, which are becoming less effective due to the development of resistance [5,6] as a result of inappropriate pesticide application practices such as the use of incorrect doses, expired products, inappropriate product selection, mixing of several pesticides together, and increased frequency of applications [7,8]. Misuse and/or overuse of insecticides may also negatively impact natural enemies of insect pests, which are useful in biological control, by causing mortality or through indirect effects such as reducing mobility, reducing growth, impairing navigation and reducing fecundity [9].

Biological control is a component of integrated pest management that refers to the reduction of a pest population by natural enemies, a process also known as natural pest regulation. Diverse insect taxa can contribute to natural pest regulation, via predation (direct consumption) or parasitism (using the pest’s body as a host for developing parasitoid young, leading eventually to the death of the pest host). Hymenoptera and, to a lesser extent, Diptera, are the major taxa of parasitoids involved in this type of interaction. Secondary parasitoids (hyperparasitoids) are those which parasitize an insect that is itself a parasitoid; these can be antagonistic to pest control as they usually kill the primary parasitoid [10], limiting the pest regulation services, but this depends upon the density of hyperparasitoids in the ecosystem [11,12].

Understanding the parasitoid community associated with aphid pests of beans can inform interventions to conserve and enhance parasitoid populations, improving biological control of insect pests. For example, the provision of floral resources in field margins or floral patches can increase the longevity and fecundity of primary parasitoids, thus increasing parasitism rates [13,14,15,16]. However, these floral resources may also enhance populations of hyperparasitoids or pests [17,18,19]; therefore, understanding interactions between pests and parasitoid guilds within the farming system is important for optimizing pest management measures. Currently, there is limited published literature on aphid parasitoid species associated with most smallholder tropical farming ecosystems. This study was carried out to identify aphid parasitoid species within the African smallholder bean farming agroecosystem in order to understand how the landscape may be modified to increase the population of primary parasitoids for biological control and whether this may be affected by secondary parasitoids.

## 2. Materials and Methods

### 2.1. Study Sites

The study sites were located at three elevations in the Kilimanjaro region of northern Tanzania within the Moshi rural district. The three zones were classified based on elevation, with the low zone defined as between 800 to 1000 m a.s.l, the mid zone between 1000 to 1500 m a.s.l and the high zone between 1500 to 1800 m a.s.l [20]. Five smallholder bean farms were selected at each elevation (farms were approximately 4100 m^2^ at low and mid elevation zones and 1100 m^2^ at the high elevation zone) and GPS coordinates for each site were recorded (Table S1). At all sites in low- and mid-elevation zones beans were grown as a monocrop during the second season (July to September), with a sunflower/maize bean intercrop having been grown at these sites the previous season (March to June). There was only one season in the high elevation zone with an intercrop farming system (beans and maize).

### 2.2. Field Sampling

Parasitoids were collected using sentinel bean plants infested with 60 ±10 aphids of mixed age (*Aphis fabae*; henceforth “sentinel aphids”) which had been reared in a greenhouse inside cages (measuring 30 × 30 × 60 cm in size with a fine mesh of <1 mm opening) to prevent any prior parasitism. Four potted sentinel plants were placed in each bean field during the major bean growing season (July to September 2017) when beans had reached the flowering stage, resulting in a total of 20 sentinel plants being placed at each elevation. Within each field, two sentinel plants were placed near the field margin (10 m apart) and two at the center of the field (10 m apart), to allow for parasitism of the sentinel aphids to take place. The aphid density on non-study bean plants in close proximity to sentinel bean plants was inspected to ensure the presence of *A. fabae*, thus increasing the likelihood that parasitoids were already present in the area. After two days of exposure, the potted bean plants were returned to cages in the greenhouse to prevent the predation of parasitized sentinel aphids. Following emergence, parasitoids were collected using an aspirator and stored at room temperature in 98% ethanol.

To determine whether the number of parasitoids was significantly different between the three elevations, negative binomial regression [21] was performed on the number of primary or secondary parasitoids that emerged from sentinel aphids in each field. A Tukey post hoc test was then done to assess the differences between elevations.

### 2.3. Parasitoid Identification

Parasitoids were identified based on morphological features using keys as described by Tomanović et al. [22]. It was possible to identify 90% of parasitoids to genus level. A representative sample of these parasitoids (74 of the 214 emerged parasitoids) was selected for molecular identification at the Natural Resources Institute, University of Greenwich, UK. DNA was initially extracted using a non-destructive method as described by Mitrović and Tomanović (2018) [23]. Where this was unsuccessful, the chelex method was used. Briefly, insects were ground individually using a plastic micropestle in 1.5 mL Eppendorf tubes containing a mixture of 90 µL chelex buffer (20% w/v chelex resine, Biorad, Hercules, California, USA, in TE solution) and 10 µL Proteinase K (ThermoFisher Scientific, Waltham, MA, USA). This mixture was then incubated at 56 °C for 20 min, then 96 °C for 5 min and finally centrifuged at maximum speed for 5 min. The supernatant was collected and stored at −20 °C. Amplification of a partial fragment of the mitochondrial cytochrome oxidase 1 (COI) gene was performed by PCR using either the LepF1 and C_ANTMRID primers [24], or the MlepF1 and LepR1 primers [25] when amplification with the first primer pair was unsuccessful. These primers were selected to give ‘mini-barcodes’ as PCR of longer barcoding regions was unsuccessful. Difficulties obtaining full-length COI barcodes have been previously documented in Hymenoptera [26] and may be due to rapid molecular evolution of the mitochondrial genome in this order [27] causing primer mismatches. It is also possible that storage of insect samples at room temperature resulted in DNA degradation or that the type of Taq DNA polymerase used was not adequate to generate full length barcodes. The ability of ‘mini-barcodes’ to differentiate between 74 or 64 parasitoid and hyperparasitoid species (LepF1/C_ANTMRID and MLepF1/LepR1 primers respectively) with COI sequences in the National Center for Biotechnology Information (NCBI) database (Tables S2 and S3) was tested using sliding window analysis [28] in Rstudio (Version 1.2.1335), with nucleotide windows of 50 bp.

The 20 µL PCR reaction mix contained 10 µL RedTaq ReadyMix (Sigma-Aldrich, St Louis, Missouri, USA), 7 µL sterile molecular-grade water, 1 µL forward primer (10 µM), 1 µL reverse primer (10 µM), and 1 µL DNA. PCR reactions were performed in a 2720 Thermal Cycler (Applied Biosystems, Foster City, California, USA) using previously described thermocycling conditions [24,25]. PCR products were visualized using electrophoresis on 1.2% agarose gels in 0.5 × TBE buffer stained with GelRed (Biotium, Fremont, California, USA). PCR products were purified using a GeneJET PCR Purification Kit (ThermoFisher Scientific, Waltham, MA, USA) following manufacturer’s instructions and sequenced by GATC Biotech (Eurofins Scientific, Luxembourg City, Luxembourg) using the forward primer (5 µM) for each gene. This produced ‘mini-barcodes’ of approximately 298 bp when amplified with LepF1/C_ANTMRID primers and 278 bp when amplified with MLepF1/LepR1 primers, which were then trimmed for analysis. These were compared to sequences in the NCBI database [29] using the Basic local alignment search tool [30].

DNA sequences of primary parasitoids amplified with LepF1/C_ANTMRID primers were used to construct a phylogenetic tree in MEGAX [31], with 8 reference sequences (Table S4) obtained from the NCBI Database [29]. The phylogenetic tree was constructed using the maximum likelihood method, based on the Tamura 3-parameter model [32] with a bootstrap value of 1000.

## 3. Results

### 3.1. Identification of Aphid Parasitoids and Hyperparasitoids Based on Morphological Features

**Figure 1:**
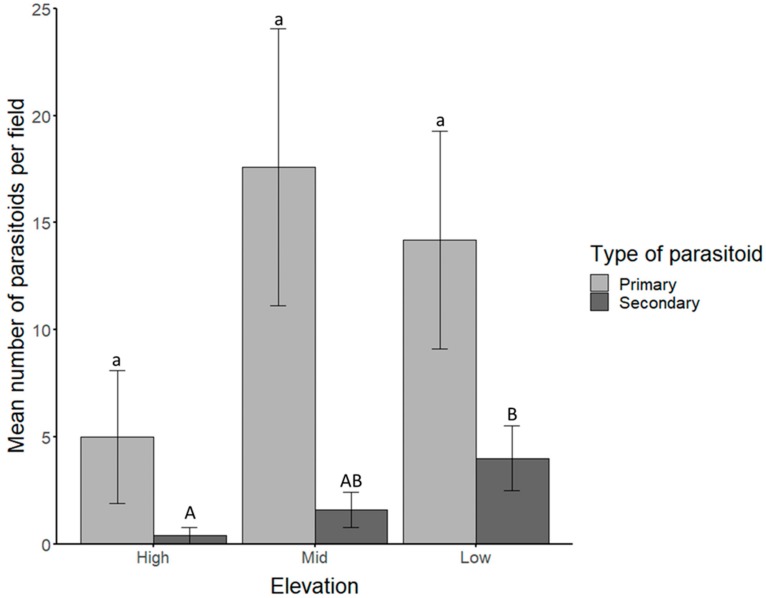
Mean numbers of primary (light gray) and secondary (dark gray) parasitoids emerged from *Aphis fabae* (Hemiptera: Aphididae) on sentinel plants in each field at high-, mid- and low-elevations. Significant differences (*p* < 0.05) are indicated by different lowercase (primary parasitoids: a) and uppercase (secondary parasitoids: **A**, **B**, **AB**) letters.

A total of 184 primary parasitoids were identified based on morphological features and isolated from the rest of the parasitoid population (Figure S1). The remaining 30 parasitoids were grouped together as secondary parasitoids until molecular identification. The highest number of primary parasitoid species were sampled from the mid-zone followed by the low-zone and the lowest number from the high-zone (Figure 1), although this difference was not statistically significant (*p* > 0.05). There was, however, a significant difference in the mean number of secondary parasitoids in each field between the high and low zone (*p* = 0.0301), but not between any other elevations (*p* > 0.05). The primary parasitoids were further identified as *Aphidius* spp. based on morphological features.

### 3.2. Identification of Aphid Parasitoids and Hyperparasitoids Based on Molecular Analysis

Sliding window analysis determined that the ‘mini-barcodes’ obtained by amplification with LepF1/C_ANTMRID and MLepF1/LepR1 primers contained suitable variation to distinguish between all the parasitoids tested. There were diagnostic nucleotides in all 50 bp windows, with the greatest sequence variation between approximately 0–50 bp and 200–250 bp for LepF1/C_ANTMRID primers and 75–125 bp for MLepF1/ LepR1 primers (Figure S2).

**Figure 2:**
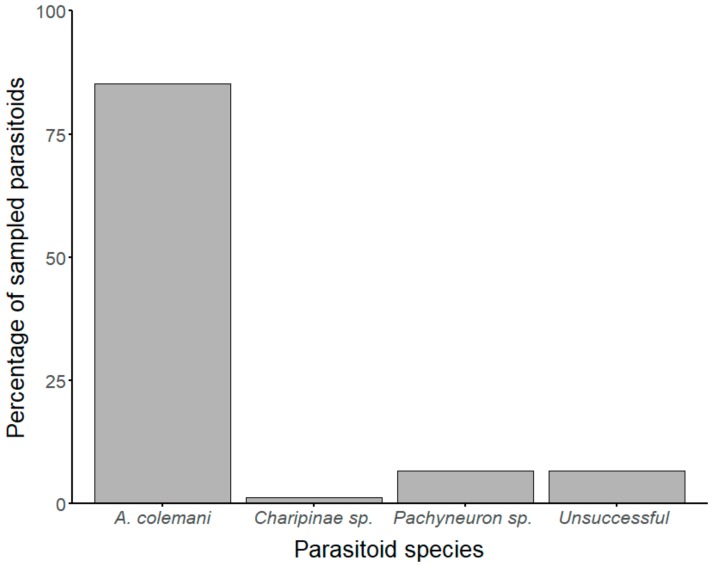
Parasitoid and hyperparasitoid species from the host *Aphis fabae* (Hemiptera: Aphididae) identified by sequencing ‘mini-barcodes’. The primary parasitoids were *A. colemani* (Hymenoptera: Braconidae: Aphidiinae). The hyperparasitoids were a *Pachyneuron* species (Hymenoptera: Pteromalidae: Pteromalinae) and a *Charipinae* species (Hymenoptera: Cynipoidea: Figitidae). The percentage of parasitoids for which sequencing was unsuccessful is also shown.

One species of primary aphid parasitoid (*Aphidius colemani* Viereck) and two species of likely hyperparasitoids (*Pachyneuron* sp. and *Charipinae* sp.) were identified through sequencing of ‘mini-barcodes’ (Figure 2). These ‘mini-barcodes’ were between 235–311 bp in length following sequence trimming and all of those used for phylogenetic tree construction were 285 bp (GenBank accession numbers in Table S5). All *A. colemani* sequences obtained from experimental samples showed ≥99% similarity to *A. colemani* sequences in the NCBI database (Table S5). All sequences from *Pachyneuron* sp. obtained from experimental samples showed 94–95% sequence similarity to *Pachyneuron aphidis* Bouché sequences from the NCBI database. These samples likely belong to the genus *Pachyneuron* but could not be identified to species level. The other sequence showed 90% similarity to a *Charipinae* sp. in the NCBI database, therefore the sample may be a closely related species, possibly in the same subfamily (Hymenoptera: Cynipoidea: Figitidae: Charipinae).

**Figure 3:**
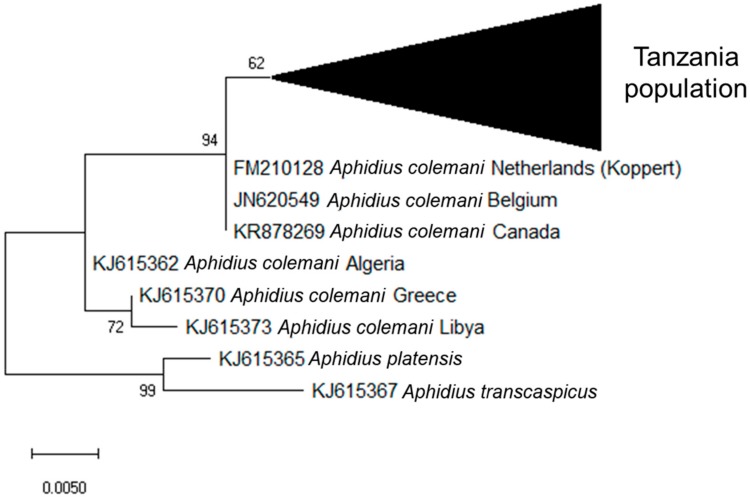
Phylogenetic tree for *Aphidius* spp. based on a 285 bp portion of the cytochrome oxidase I gene. The tree was constructed using the maximum likelihood method based on the Tamura 3-parameter model [32] with a bootstrap value of 1000.

The 55 *A. colemani* from this study sequenced using LepF1/C_ANTMRID primers are closely related, but not all identical, with a mean of 0.007 base substitutions per site (Figure 3). They appear to cluster with *A. colemani* from the Netherlands (Koppert Biological Systems, Berkel en Rodenijs, Netherlands), Belgium, and Canada, with a mean of 0.008 base substitutions between these groups. The *A. colemani* population from Tanzania shows more divergence from *A. colemani* in Algeria, Greece and Libya (Mediterranean populations) with a mean of 0.022 base substitutions per site between these groups.

## 4. Discussion

The study found the primary parasitoid of bean aphids, *A. fabae*, within the smallholder bean farming tropical ecosystem to be *A. colemani*, which was accompanied by two species of secondary parasitoids (*Pachyneuron* sp. and *Charipinae* sp.). The number of primary parasitoids was not significantly different between elevations; however, there were more secondary parasitoids at fields in the lowest elevation compared to the highest elevation. Further studies would need to be conducted to conclusively determine the effect of elevation and intercropping on the parasitoid community. The genus *Aphidius* contains more than 70 species of aphid parasitoids, which have a wide distribution [33]. *A. colemani* is a solitary endoparasitoid known to parasitize several species of economically important aphids including *Aphis fabae*, *Aphis gossypii*, *Rhopalosiphum padi*, and *Myzus persicae* [34,35]. It has been widely used in biological control programs since the 1970s [36] and is reared on a commercial scale for aphid control in more than twenty countries [34]. Despite the widespread use of *A. colemani*, there remains uncertainty regarding the classification of this species [37]. Historically, *Aphidius transcaspicus* Telenga and *Aphidius platensis* Brethes have been misidentified as *A. colemani* [38] due to morphological similarities and overlapping distributions [22]. DNA barcoding has since been used to confirm that these species are genetically distinct [22,37], which is supported by the findings of this study. However, it has been reported that despite being genetically determined to be different species, *A. transcaspicus* and *A. colemani* are able to mate and produce fertile offspring, suggesting that *A. colemani* might be a complex of different species [37]. Further sequencing and cross mating studies may be needed to verify the relationship between these two species.

The *A. colemani* characterized in this study appeared most closely related to those available commercially (Koppert, Berkel en Rodenrijs, the Netherlands), which have become established in a number of countries following commercial use in glasshouses [39,40]. In general, *A. colemani* is thought to originate from India or Pakistan [41]; however. the origin of this commercial strain is uncertain. It has been reported that a population of *A. colemani* (originally identified as *Aphidius* sp.) was collected in East Africa, then propagated at the Commonwealth Institute of Biological Control (CIBC) Pakistan station. This population was then transported to the U.K for biological control of *Myzus persicae*; from here it may have spread to other countries, including the Netherlands, where the Koppert strain originated [39]. While the results of this study may lend support to this ‘Out of Africa’ hypothesis on the origin of the commercially available *A. colemani*, it is also possible that the *A. colemani* population sampled here was a result of an earlier, unrecorded release of the Koppert strain in the study region, as has been documented in other countries [39,40]. In order to conclusively determine the origin of the commercial strain, historical specimens of *A. colemani* from East Africa (prior to the use of the commercial strain) would need to be sequenced. Further taxonomic study is also needed to confirm the taxonomic status of the *A. colemani* population in Tanzania due to variability in the COI barcoding region in comparison with Mediterranean populations.

Characteristics which make *A. colemani* an effective biocontrol agent include high dispersal distance and searching ability [42]. Indeed, *A. colemani* can be as effective as a synthetic pesticide (imidacloprid) in managing aphid population in greenhouse conditions [35], signifying the potential of this parasitoid wasp in aphid control. The efficiency of *A. colemani* can be affected by both biotic and abiotic factors, with hyperparasitism an important biotic factor since it affects the abundance of the primary parasitoids as well as modifying their behavior [36]. Some of the reported behavioral changes include abandonment of the patches by the primary parasitoid females in the presence of hyperparasitoids, regardless of aphid density, in order to minimize the mortality rate of their progeny [43,44]. This means at a high hyperparasitoid density, there is more dispersal of the primary parasitoids from the patches without complete exploitation of the aphids. It is possible that aphids have evolved mechanisms to attract secondary parasitoids, indeed some secondary parasitoids are attracted by the volatiles from aphid honeydew [45]. Aphid reproduction also increases in the presence of volatile chemicals released from secondary parasitoids without physical contact in the field, suggesting that aphids respond to chemical cues from secondary parasitoids [10,46]. However, there is a need for more field experiments to investigate the multitrophic interactions within the aphid/parasitoid system over time and evaluate the possible consequences on pest control.

The level of hyperparasitism of *A. colemani* in agricultural systems ranges from low to very high and may reach 100% [47]. In our study the low elevation zone had a high number of hyperparasitoids compared with the high zone but this may vary depending on cropping season; hyperparasitism was found not to compromise aphid control during the spring season in the Netherlands, whereas in summer, the aphid control failed completely due to hyperparasitism [11]. The factors influencing the ratio of primary to secondary parasitoids are not well understood, but some indications are that, as for other functional groups, hyperparasitoid species richness may be affected by landscape complexity [48], and agricultural intensification may increase the rate of secondary relative to primary parasitism [49]. Hyperparasitoids may pose a short-term risk to biological control of aphids, though it is reported that a stable equilibrium consisting of aphids, parasitoids and hyperparasitoids may be beneficial for long term biological control if the system is at sub economic level [50]. Therefore, when considering primary parasitoids for biological control, it is important to understand the long-term influence of hyperparasitoids and how they may affect the ability of the primary parasitoid to control the target pest.

The fact that *A. colemani* was the only primary parasitoid detected in the field indicates that it is the most immediate candidate for aphid control in these smallholder systems and suggests that biological control of aphids here should thus focus on providing resources that support high *A. colemani* abundance year-round. This could particularly include food (nectar) resources: Previous studies identified plants including *Fagopyrun esculentum*, *Salvia apiana*, *Lantana camara*, and *Conium maculatum* as having positive effects on parasitoid longevity and parasitism rates [51,52]. However, these plant species are non-native in northern Tanzania and, as such, are unsuitable for promotion in smallholder East African farming systems, and there has not been sufficient research into the effect they may have on the other trophic levels within these ecosystems. Therefore, more research is needed to determine the best floral resource plants to augment *A. colemani* populations in the smallholder bean farm tropical ecosystem and to assess their impact on hyperparasitoids, particularly *Pachyneuron* sp., and pest species.

## 5. Conclusions

*A. colemani* is an important biological control agent in the management of aphids at a commercial scale. Historically, there has been less effort made to integrate biological control into agriculture in most African countries, despite the abundance of information about biological control programs implemented in Europe and America. This study focused on characterizing the parasitoid community of an East African smallholder bean farming system in order to assess the impact of biological control of aphids. The information on the parasitoid and hyperparasitoid community is useful for developing farming practices, such as promotion of floral resource plants, to increase the efficacy of parasitoids for aphid control. We found variation in hyperparasitism levels between agro-ecological zones, indicating continuous monitoring of the hyperparasitism levels is needed. It is important to characterize the dynamics of aphids, primary parasitoids and secondary parasitoids in different zones and cropping seasons, and the implications for aphid control. As *A. colemani* was identified the dominant parasitoid of bean aphids in these systems, it is recommended as a target for future conservation biological control programs. Future research should identify plants that increase fecundity and survival of *A. colemani* in these systems, while not benefitting hyperparasitoid and pest species.

## Supplementary Materials

The following are available online at https://www.mdpi.com/2075-4450/10/10/331/s1, Figure S1: Parasitoids emerged from sentinel aphids, Figure S2: Sliding window analysis for LepF1/C_ANTMRID and MLepF1/LepR1 mini-barcodes, Table S1: Location of field sites, Table S2: Sequences from the NCBI database used to perform sliding window analysis for LepF1/C_ANTMRID primers, Table S3: Sequences from the NCBI database used to perform sliding window analysis for MLepF1/LepR1 primers, Table S4: Sequences from the NCBI database used to construct phylogenetic tree and Table S5: BLAST matches and accession numbers of parasitoids analyzed in this study.
