# Supplementary material for: Characterization of Hymenopteran Parasitoids of Aphis fabae in An African Smallholder Bean Farming System Through Sequencing of COI ‘Mini-barcodes’
Source: Insects. 2019 Oct 2;10(10):331. doi: 10.3390/insects10100331 (PMC6835700; doi:10.3390/insects10100331)
Supplement: Supplementary file 1 [file insects-10-00331-s001.zip › Supplementary Information/Figures S1 and S2.pdf]

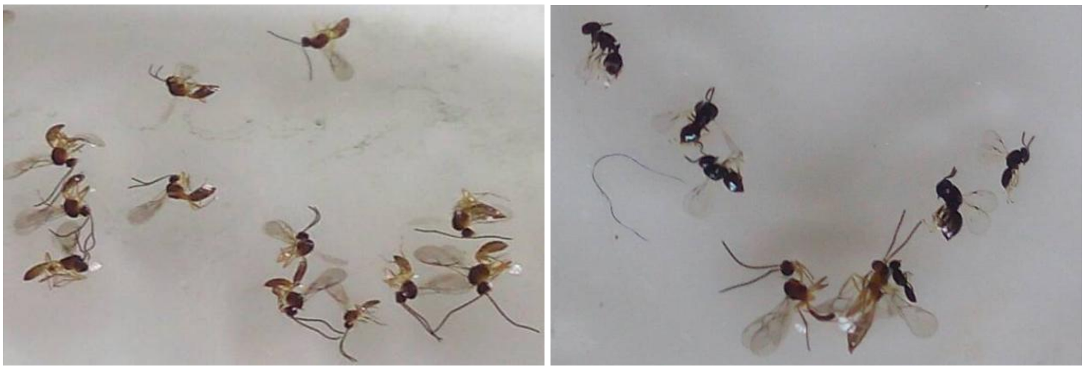

Figure S1. Parasitoids emerged from sentinel aphids. Parasitoids emerged from *Aphis fabae*, viewed under a stereo microscope (magnification x25).

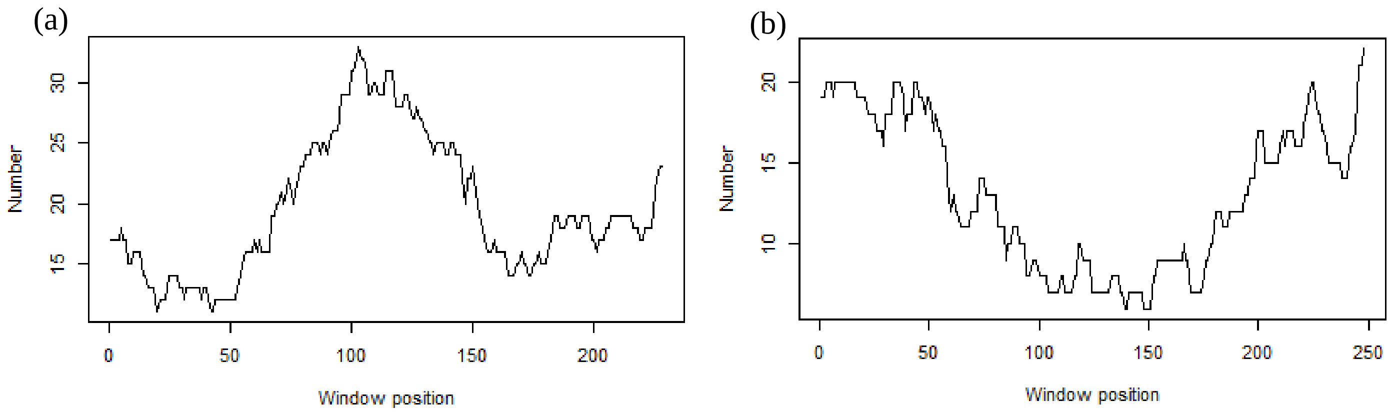

Figure S2. Sliding window analysis for LepF1/C_ANTMRID and MLepF1/ LepR1 mini-barcodes. Sum of diagnostic nucleotides in 50 bp windows for mini-barcodes LepF1/C_ANTMRID (a) and MLepF1/ LepR1 (b), when comparing nucleotides from 74 or 67 parasitoid species, respectively (Tables S2 and S3).
